# Peroxisome Proliferator-Activated Receptor Genetic Polymorphisms and Nonalcoholic Fatty Liver Disease: Any Role in Disease Susceptibility?

**DOI:** 10.1155/2013/452061

**Published:** 2013-02-04

**Authors:** Paola Dongiovanni, Luca Valenti

**Affiliations:** Section of Internal Medicine, Department of Pathophysiology and Transplantation, Università degli Studi di Milano, Fondazione IRCCS Ca' Granda Ospedale Maggiore Policlinico, Pad. Granelli, Via F Sforza 35, 20122 Milano, Italy

## Abstract

Nonalcoholic fatty liver disease (NAFLD) defines a wide spectrum of liver diseases that extend from simple steatosis, that is, increased hepatic lipid content, to nonalcoholic steatohepatitis (NASH), a condition that may progress to cirrhosis with its associated complications. Nuclear hormone receptors act as intracellular lipid sensors that coordinate genetic networks regulating lipid metabolism and energy utilization. This family of transcription factors, in particular peroxisome proliferator-activated receptors (PPARs), represents attractive drug targets for the management of NAFLD and NASH, as well as related conditions such as type 2 diabetes and the metabolic syndrome. The impact on the regulation of lipid metabolism observed for PPARs has led to the hypothesis that genetic variants within the human PPARs genes may be associated with human disease such as NAFLD, the metabolic syndrome, and/or coronary heart disease. Here we review the available evidence on the association between PPARs genetic polymorphism and the susceptibility to NAFLD and NASH, and we provide a meta-analysis of the available evidence. The impact of PPAR variants on the susceptibility to NASH in specific subgroup of patients, and in particular on the response to therapies, especially those targeting PPARs, represents promising new areas of investigation.

## 1. Introduction

Nonalcoholic fatty liver disease (NAFLD), a major cause of progressive liver disease, is defined by an increase in hepatic fat content not related to toxics and has a strong genetic component. As the peroxisome proliferator-activated receptors (PPARs) represent major regulators of lipid metabolism in the liver, a few studies have tested the hypothesis that genetic variants in these hormone receptors may influence the susceptibility to NAFLD, but with controversial results. In this paper, we provide an overview of the published evidence in the field, and a meta-analysis of the available results on the role of the Pro12Ala PPAR*γ* single nucleotide polymorphism (SNP), the most studied genetic variant to date. As PPARs are also the target of several drugs under evaluation for the treatment of NAFLD, this evidence may lay the basis to design pharmacogenetic studies to assess the role of PPARs SNPs in predicting the response to drugs targeting these nuclear receptors.

## 2. Nonalcoholic Fatty Liver Disease (NAFLD)

Liver fat deposition related to systemic insulin resistance (IR) defines NAFLD [1]. The acronym NAFLD defines a wide spectrum of liver disease ranging from simple uncomplicated hepatic fat accumulation in the form of triglycerides exceeding 5% of liver mass in the absence of significant alcohol consumption to severe steatohepatitis characterized by severe steatosis, lobular inflammation, and hepatocellular damage and apoptosis with the activation of fibrogenesis [2], which can progress to cirrhosis and hepatocellular carcinoma [3]. Due to the epidemic of obesity and the metabolic syndrome, NAFLD is now the most frequent liver disease (prevalence 20–34%) and the leading cause of altered liver enzymes in Western countries [4, 5]. Hepatic fat accumulation results from an unbalance between triglycerides acquisition and removal [6] and initially represents a protective mechanism to shield hepatocytes from the toxicity resulting from an increased flux of free fatty acids (FFAs) to the liver [7]. Several lines of evidence support the hypothesis that most of the FFAs accumulated as triglycerides during steatosis derive from increased peripheral lipolysis [8] related to adipose tissue IR [9], followed by increased lipogenesis induced by hyperinsulinemia and diet. Indeed, the major risk factor for NAFLD is represented by systemic IR related to central obesity and the metabolic syndrome [1, 10]. Steatosis *per se* may then precipitate hepatic IR contributing to metabolic disturbances and cardiovascular damage [11, 12]. Impaired ability to secrete lipoproteins [13] and decreased *β*-oxidation due to mitochondrial damage (in particular in the presence of NASH) may also play a role in hepatic fat accumulation.

Epidemiological, familial, and twin studies have recently provided clear evidence for an element of heritability of NAFLD [14–16]. During the last years, genetic modifiers of disease severity and progression have been identified through genome-wide association studies [17, 18]. These include the Patatin-like phospholipase domain-containing 3 (PNPLA3) I148M gene variant, which has been demonstrated to represent a major determinant of interindividual and ethnicity-related differences in hepatic fat content independent of IR and serum lipid concentration, and a determinant of the progression towards NASH and fibrosis [19, 20]. Furthermore, a few large multicenter case-control studies demonstrated a role of SNPs implicated in insulin signalling [21], oxidative stress [22], and fibrogenesis [23] in the progression of NAFLD towards NASH confirming that hepatocellular fat accumulation and IR are key operative mechanisms in the pathophysiology of NAFLD and are closely involved in the progression of liver damage. New genetic risk factors could prove useful for the clinical management of patients with NAFLD and for the identification of novel therapeutic targets for NASH, for which specific treatments are still lacking.

## 3. Peroxisome Proliferator-Activated Receptors: The PPARs

The PPARs represent novel targets for the development of therapeutic agents for the treatment of metabolic syndrome, obesity, dyslipidemia and type 2 diabetes. Nuclear receptors are transcription factors that serve as intracellular receptors for endocrine hormones and dietary lipids. Differently from extracellular receptors which bind to peptide ligands and activate cytoplasmic kinase cascades, nuclear receptors interact directly with lipophilic ligands and regulate the expression of target genes [26]. These receptors can be considered the body's lipid sensor that can monitor the concentration of bioactive lipids and coordinate the enzymatic cascades that regulate lipid synthesis and utilization. There are three members of the PPAR family each encoded by a different gene: PPAR*α* (NR1C1), PPAR*γ* (NR1C3), and PPAR*δ* (NP1C2). All three PPARs bind to DNA as heterodimers with the retinoid X receptor (RXR).

## 4. PPAR**α**

PPAR*α* directly regulates a network of genes encoding protein involved in fatty acids uptake, enzymes required for the oxidation of fatty acids (*β*-oxidation), and enzymes required for ketogenesis by binding to control regions in the promoter of these genes and by promoting fat utilization [27]. The net effect is increased fatty acids oxidation, decreased serum triglycerides, and an increase in cholesterol efflux. PPAR*α* is predominantly expressed in tissues capable of oxidizing fatty acids such as liver, heart, muscle, brown adipose tissue, and the kidney. PPAR*α* can be activated by natural lipophilic ligands such as fatty acids and by drugs approved for the treatment of hypertriglyceridemia, such as fibrates [28]. The role of PPAR*α* in the pathogenesis of fatty liver became evident in PPAR*α* KO mice. These mice are unable to upregulate fatty acid catabolism and develop steatosis, myocardial lipid accumulation, and hypoglycaemia during short-term starvation or after high-fat diet [29, 30]. Taken together, mouse models suggest that PPAR*α* functions to increase fatty acid use in the fasting state, and that in the context of a high-fat diet PPAR*α*, inducing fatty acid catabolism, might prevent hepatocellular fat accumulation and hypertriglyceridemia. PPAR*α* downregulation is involved in NASH pathogenesis by reducing FFA catabolism [31]. 

## 5. PPAR**α** Polymorphisms and NAFLD

The role of PPAR*α* gene polymorphisms in NAFLD and the regulation of lipid metabolism has been investigated in a few studies. Chen et al. hypothesized that the coding Val227Ala SNP in the PPAR*α* gene may be implicated in the pathogenesis of NAFLD. In 79 NAFLD patients and 63 healthy controls, it was found that the PPAR*α* Val227Ala genotype frequency was significantly different between NAFLD and control subjects and that the fat-related index such as weight, body mass index (BMI), hip circumference, waist circumference, waist-to-hip ratio, and the percentage of body fat of the carriers of the Ala227 allele was lower than that in noncarriers [24]. Yamakawa-Kobayashi et al. evaluated the Val227Ala SNP in 401 healthy Japanese subjects. Total cholesterol was lower in Ala227 carriers than in noncarriers, and the lipid profiles of Ala227 carriers appeared favourable compared with those of non-carriers. Since the Val227Ala variant is located in the region between the DNA-binding and ligand-binding domains, which is also thought to contain the dimerisation domain of the protein, it has been hypothesized that the substitution of Valine to Alanine at codon 227 causes a functional change in PPAR*α*, and that the Ala227 isoform has higher activity than the Val227 isoform [32], thus leading to enhanced ability to burn fatty acids, and potentially explaining the association with lower lipid levels and the protection from steatosis development. However, Sparsø et al. genotyped the Leu162Val SNP in the PPAR*α* gene in 5799 middle-aged white people, but they did not find any association with obesity or type 2 diabetes. Though, another PPAR*α* coding polymorphism, the Leu162Val variant, was possibly associated with increased fasting cholesterol and triglyceride concentrations [33]. The relationship between the Leu162Val SNP and NAFLD was further evaluated in 202 Italian subjects compared to 346 healthy controls. The frequency of this SNP did not differ between patients and controls, but the presence of the PPAR*α* 162Val allele was associated with higher IR, but not histologically assessed disease severity [25], suggesting that the risk related to increased IR may be balanced by the protective effect of decreased oxidative stress, the other key player in the progression of liver disease in patients with NASH. Results of the published association studies between PPAR*α* polymorphisms and NAFLD are summarized in Table 1.

## 6. PPAR**γ**

PPAR*γ* is the master regulator of adipogenesis and plays an important role in the process of lipid storage [34]. PPAR*α* and PPAR*γ* have therefore opposing functions in the regulation of fat metabolism; PPAR*α* promotes fat utilization while PPAR*γ* promotes fat storage. PPAR*γ* is expressed in adipocytes, macrophages, and muscle, where it regulates development, lipid homeostasis, and glucose metabolism. Mice lacking PPAR*γ* are embryonically lethal, but the development of conditional PPAR*γ* knockouts has confirmed the essential role of PPAR*γ* in adipocytes differentiation and survival [35]. Moreover, specific deletion of PPAR*γ* in fat and muscle causes IR underlying its importance in peripheral insulin sensitivity [36]. Possible mechanisms underlying the insulin sensitizing activity of PPAR*γ* include increased lipid uptake and storage leading to decreased free fatty acids and serum triglycerides, induction of the expression of adiponectin, a molecule with anti-inflammatory and insulin-sensitizing effect by adipocytes [37], and the suppression of hepatic gluconeogenesis and increased glucose uptake by adipose tissue through GLUT4 upregulation [38]. Fatty acids and prostanoids act as PPAR*γ* agonists. However, also the thiazolidinediones (TZDs), a class of insulin sensitizers that are approved for the treatment of type 2 diabetes and have been shown to decrease steatosis in patients with NASH [39, 40], function as high affinity PPAR*γ* agonists. The activation of PPAR*γ* by TZDs induces the expression of a set of genes involved in adipocytes differentiation and lipogenesis and induces adiponectin, thus explaining the insulin-sensitizing action of these drugs [41, 42].

## 7. Role of PPAR**γ** Polymorphisms: Rationale and Available Studies

Human genetics has provided evidence of a role of PPAR*γ* in the metabolic syndrome [43]. Dominant negative mutations in PPAR*γ* are the cause of a monogenic disease characterized by severe insulin resistance, type 2 diabetes, and hypertension [44]. Importantly, a frequent coding SNP in the PPAR*γ* gene, the Pro12Ala variant, has consistently been associated in metabolic studies with BMI, insulin sensitivity, the metabolic syndrome [45]. The N-terminal proline to alanine exchange (Pro12Ala) occurs in the extra domain of the PPAR*γ*2 transcript: this PPAR*γ* splice isoform includes 30 additional amino acids [46], which are responsible for an increase of PPAR*γ* transcriptional activity in the adipose tissue. The Pro12Ala exchange results from a cytosine to guanine substitution in the PPAR*γ* gene, encoding the Ala allele form with a strongly reduced function [47]. The association between the positivity for the 12Ala variant and IR, type 2 diabetes, higher BMI, and obesity has already been well described and confirmed in several studies [48–50]. This association may be explained by the lower activity of the 12Ala variant in the adipose tissue, favouring IR and potentially the flux of FFAs to the liver and NAFLD. However, the role of this SNP in the pathogenesis and progression of fatty liver disease is still debated. 

Rey et al. analyzed the presence of the Pro12Ala polymorphism in 622 German Caucasian subjects suffering from fatty liver (263 NAFLD patients and 100 with alcoholic fatty liver disease (AFLD) subjects) or being healthy blood donors (*n* = 259). In fatty liver disease patients the Ala allele was more represented than in controls. In NAFLD patients the higher prevalence of the 12Ala allele was not associated with the progression of liver disease, whereas AFLD patients carrying the 12Ala allele had a higher risk of severe steatohepatitis and fibrosis [53]. Similarly, the 12Ala allele was not associated with NAFLD susceptibility, liver damage, or IR in 212 Italian patients with NAFLD [25]. Gupta et al. analyzed the genotype frequencies of the Pro12Ala variant in 98 NAFLD patients and 280 matched controls and found a higher prevalence of heterozygosity for the Ala variant in patients. Moreover, in NAFLD patients the 12Ala variant was also associated with overweight (BMI > 25 Kg/m^2^), suggesting an important role of Pro12Ala variant in the obesity-related NAFLD disease pathogenesis [52]. Gawrieh et al. investigated the association between two PPAR*γ* variants (the Pro12Ala and a second common SNP, the C1431T) with NAFLD and its histological features. They considered 212 patients with NAFLD and 63 controls and found that individual SNPs did not show significant association with NAFLD. The haplotype defined by the presence of both minor alleles (GT) was less enriched, whereas an haplotype, comprised of the two major alleles (CC), was more enriched in subjects with NAFLD compared to controls, and both haplotypes were significantly associated with steatosis and fibrosis [51]. As the carriers of the 12Ala variant have been reported to have increased resistance to oxidative stress [55], and since smoking increases the production of reactive oxygen species, Yang et al. explored the influence of the Pro12Ala SNP on the risk of NAFLD and determined whether this polymorphism and smoking showed a synergistic effect on the development of NAFLD in middle-aged and older Chinese people (considering 436 NAFLD patients and 467 controls). The 12 Pro/Pro genotype and smoking were significant independent risk factor for NAFLD. In addition, the higher risk group (smokers with the 12 Pro/Pro genotype) showed 3.75 times higher risk of NAFLD than the low-risk group (nonsmokers with the 12 Pro/Ala genotype). However, no relationship between the PPAR*γ* gene and grading for steatohepatitis was observed. They hypothesized a possible synergistic effects of genotype and smoking in the development of NAFLD by aggravating oxidative stress [54]. Zhou et al. investigated the association of seven candidate SNPs with susceptibility to NAFLD in 117 Chinese patients and matched controls and found that the genotypic distributions and allelic frequencies of the PPAR*γ* gene −161 C/T polymorphism in the NAFLD group were significantly different from those in the control group suggesting that the C/T variant increased the susceptibility to NAFLD [56]. Finally, very recently Bhatt et al. have investigated the associations of polymorphisms C161T and Pro12Ala of PPAR*γ* with clinical and biochemical parameters in 162 Asian patients with ultrasonographically diagnosed NAFLD and 173 controls. They found that the Pro12Ala polymorphism was associated with significantly higher serum TG, alkaline phosphatase, and waist-hip ratio, whereas the C161T polymorphism with increased TG and total cholesterol. At multivariate analysis, NAFLD was associated with these two polymorphisms [57].

## 8. A Meta-Analysis of Available Studies on the Association between PPAR Pro12Ala Variant and NAFLD

**Figure 1:**
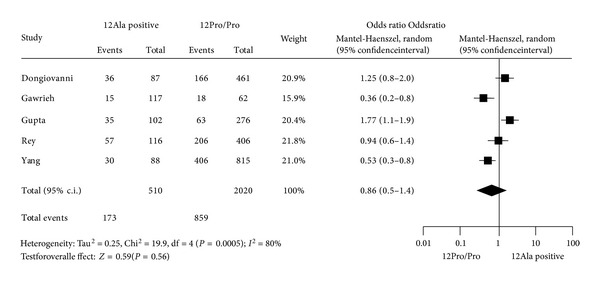
Meta-analysis of the effect of the Pro12Ala PPAR*γ* variant on the risk of NAFLD in published studies. The odds ratio (ORs) and the corresponding 95% confidence interval (c.i.) limits (lower and upper) are calculated by random effects meta-analysis (Mantel-Haenszel; M-H) for nonalcoholic fatty liver disease (NAFLD) according to the Pro12Ala variant (12Ala positive versus 12Pro/Pro genotype). In the “Study” is cited the first author of the study. “Events” indicate the number of patients with NAFLD who carry a genotype (e.g., 36 positive for 12Ala allele) while “Total” indicates the sum of patients and controls with the same genotype (e.g., 87 is the sum of NAFLD patients and controls who carry the 12 allele). In the graph, numbers indicate OR and filled diamond express random effect. The symbol size is proportional to the weight of the study.

In view of the still controversial evidence concerning the association between PPAR*γ* genotype and NAFLD susceptibility mentioned above, we decided to estimate from the available literature the strength of the effect of Pro12Ala variant of PPAR*γ* gene on NAFLD across different populations. In contrast, due to the heterogeneity of genetic markers evaluated in previous studies, it was not possible to conduct a meta-analysis of PPAR*α* studies. For the electronic searches, published studies were found through PubMed at the National Library of Medicine (http://ncbi.nlm.nih.gov/entrez/query/) for the query NAFLD, PPAR*γ* polymorphism, and Pro12Ala variant (rs1801282). References list in relevant publications was also considered. The literature search was done on studies up to 2012, written in English and for which were available abstracts and complete article. For the meta-analysis we considered five papers, which are presented in Table 2. There were not country restrictions. The presence of NAFLD was diagnosed by biopsy or ultrasound. All the studies were population-based case-control studies. Complete or partial information about liver biopsy was available in four studies, and data about fatty liver was analyzed in 1238 subjects with NAFLD. Genotyping for rs18012282 was carried out using TaqMan allelic discrimination in three studies [52–54] and by polymerase chain reaction (PCR) and restriction analysis in two studies [25, 52]. The calculations were performed using the free meta-analysis REV Manager 5.0 Software Informer (http://ims.cochrane.org/revman/). Results of the meta-analysis are presented in Figure 1. This meta-analysis, by summarizing the amount of evidence, failed to detect a significant association between the Pro12Ala SNP in the PPAR*γ* gene and NAFLD, highlighting at the same time a significant heterogeneity among the published studies. 

In line with this result, no genome-wide association studies found an association between genomic variants in PPARs genes and NAFLD. Moreover, the majority of studies indicate that the Pro12Ala variant is especially involved in the development of type 2 diabetes. However, it is possible that the Pro12Ala polymorphism in the PPAR*γ* gene may contribute to the pathogenesis of NAFLD in the presence of other genetic variants or in the presence of environmental risk factors, such as obesity. Therefore, future studies should be conducted in larger series of well-characterized patients with a homogenous clinical subphenotype of NAFLD (e.g., in severely obese patients) and should be controlled for other major risk factors for NAFLD, such as the I148 M PNPLA3 variant. In conclusion, the Pro12Ala variant cannot be considered a clinically relevant marker for NAFLD, at least when evaluated alone in the overall population. Despite this, as we are moving towards individualized medicine, these data could provide the basis to design pharmacogenetic studies to address whether therapeutic efficacy of PPAR*γ* agonists in NAFLD patients is affected by the 12Ala SNP alone or in combination with other SNPs of genes involved in lipid metabolism, and whether PPAR SNPs may modify NAFLD risk in specific populations.

## 9. PPAR**δ**

PPAR*δ* is ubiquitously expressed, most highly in brain, macrophages, lung, adipose tissue, and skeletal muscle [26, 58] and is activated by fatty acids and components of very-low-density lipoprotein (VLDL) [28, 59]. PPAR*δ* activation enhances fatty acids transport and oxidation, improves glucose homeostasis via the inhibition of hepatic glucose output, reduces macrophages inflammatory responses, and increases HDL levels [60]. PPAR*δ* knockout mice die in midgestation. Surviving mice show markedly decreased adipose tissue suggesting a requirement for PPAR*δ* in peripheral tissues [61]. Further support for a role of PPAR*δ* in lipoprotein metabolism results from studied exploring the activity of the PPAR*δ* specific synthetic agonist GW501516. Treatment of animals including primates with GW501516 significantly increases HDL, lowers triglycerides, and LDL and decreases fasting insulin levels [62, 63]. Synthetic PPAR*δ* agonists have proven to be effective also in preclinical model of diabetes and dyslipidemia, and preliminary results are also available for steatosis. Results of a two-week phase II study in patients with dyslipidemia demonstrated that total cholesterol, LDL cholesterol, triglycerides, and nonesterified fatty acids were significantly lowered by GW501516 [64]. The impact on the regulation of lipid and carbohydrate metabolism observed for PPAR*δ* has led to the hypothesis that genetic variation within the human PPAR*δ* gene may be associated with human disease such as the metabolic syndrome and/or coronary heart disease. The +294 T/C polymorphism in exon 4 of the PPAR*δ* gene seems to influence binding of Sp-1 resulting in higher transcriptional activity for the rare C allele than the common T allele [65]. Skogsberg et al. observed in 543 healthy, middle-aged men that the C genotype was associated with elevated levels of LDL cholesterol and ApoB [66]. In 580 male subjects with hyperlipidemia recruited from the West of Scotland Coronary Prevention Study (WOSCOPS) carriers of the C allele had significantly lower HDL plasma concentrations [67], whereas Aberle et al. found a highly significant association between the rare C allele and lower plasma HDL concentrations in 967 females with mixed hyperlipidemia [68]. Robitaille et al. identified 15 variants in the PPAR*δ* gene and found that another polymorphism (−87 T > C) was associated with a lower risk to exhibit the metabolic syndrome and that this association was influenced by dietary fat intake [69]. Andrulionyte et al. found that SNPs of the PPAR*δ* gene may modify the conversion from IGT to type 2 diabetes particularly in combination with Gly482Ser SNP of the PPAR*γ* coactivator-1A (PGC-1A) and Pro12Ala SNP of PPAR*γ*2 [70]. Grarup et al. investigated variation in PPAR*δ* gene in 6071 Danish white subjects of whom 4543 had NGT, 503 had IFG, 693 had IGT, and 352 had diabetes. They concluded that common variation in PPAR*δ* does not affect the risk of metabolic disease in the population studied [71]. However, no published study specifically addressed the role of PPAR*δ* SNPs in the susceptibility to NAFLD.

## 10. Conclusions

Available studies do not provide sufficient evidence for a significant evidence for an association between PPAR*α* and PPAR*γ* SNPs, and the risk of NAFLD. In particular, our meta-analysis of the effect of the Pro12Ala PPAR*γ*2 SNP, the best studied genetic factor to date, and NAFLD did not provide conclusive results. However, most of the studies were underpowered, the definition of the NAFLD phenotype was rather heterogeneous (histological versus ultrasonographic versus based on liver enzymes), the analyses were conducted in ethnically diverse population, and most studies were not controlled for other genetic risk factor for NAFLD such as the PNPLA3 I148 M SNP, so that the patients included were not phenotypically homogeneous. Furthermore, even scarcer data are available for the association of PPARs variant with the progression of liver damage, and variants of PPAR*δ*, another nuclear receptor involved in IR and lipid metabolism, were not assessed. Most importantly, PPARs are promising targets for NASH, but no study has yet assessed the effect of genetic variants in PPARs genes and the effect of therapy.

The evaluation of the impact of PPAR variants on (1) the susceptibility to NASH in specific subgroup of patients such as severely obese subjects in adequately powered studies and (2) on the response to drugs targeting PPARs (such as glitazones or PPAR*α*/*δ* agonists, which are under study in NASH patients), represent promising new areas of investigation.

## Figures and Tables

**Table 1:** Characteristics of the studies on the association between the PPAR*α* polymorphisms and nonalcoholic fatty liver disease.

First author, year	Ref.	PPAR*α* variant	Population ethnicity, country	Sample size (*N*)	Patients characteristics	Liver biopsy (*N*)	Female sex, *N* (%)	Conclusions
Chen, 2008	[24]	Val227Ala	China	*N* = 79	Unspecified NAFLD	*N* = not specified	40 (51)	Association with NAFLD
Dongiovanni, 2010	[25]	Leu162Val	Caucasian, Italy	*N* = 202	Histological NAFLD	*N* = 202	41 (20)	No association with NAFLD

Ref: reference number; *N*: number; NAFLD: nonalcoholic fatty liver disease.

**Table 2:** Characteristics of the studies on the association between the Pro12Ala variant of PPAR*γ* and nonalcoholic fatty liver disease.

First author, year	Ref.	Ethnicity, country	Study design, sample size (*N*)	Patients characteristics	Liver biopsy (*N*)	Female sex, *N* (%)
Dongiovanni, 2010	[25]	Caucasian, Italy	Case-control *N* = 202	Histologically proven NAFLD	*N* = 202	41 (20)
Gawrieh, 2011	[51]	Caucasian, USA	Case-control *N* = 212	Histologically proven NAFLD	*N* = 212	145 (68)
Gupta, 2011	[52]	Asian, India	Case-control *N* = 98	Diagnosis based on ultrasound	*N* = 71	32 (33)
Rey, 2010	[53]	Caucasian, Germany	Case-control *N* = 263	Histologically proven NAFLD	*N* = 263	Not specified
Yang, 2012	[54]	Asian, China	Case-control *N* = 436	Diagnosis based on ultrasound	—	280 (64)

Ref: reference number; *N*: number; NAFLD: nonalcoholic fatty liver disease.

## Abbreviations

AFLD: Alcoholic fatty liver disease
BMI: Body mass index
FFAs: Free fatty acids
IR: Insulin resistance
NAFLD: Nonalcoholic fatty liver disease
NASH: Nonalcoholic steatohepatitis
PNPLA3: Patatin-like phospholipase domain-containing 3
PPARs: Peroxisome proliferator-activated receptors
RXR: Retinoid X receptor
SNP: Single nucleotide polymorphism
VLDL: Very low-density lipoproteins.
